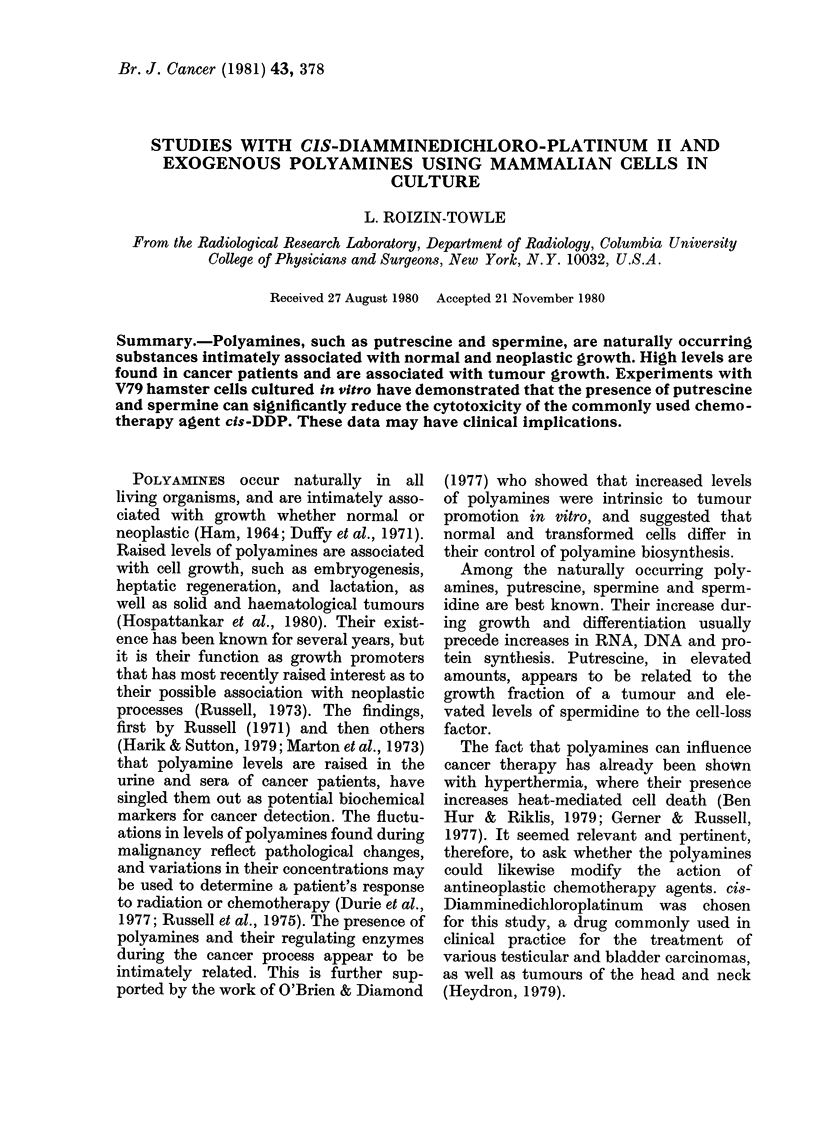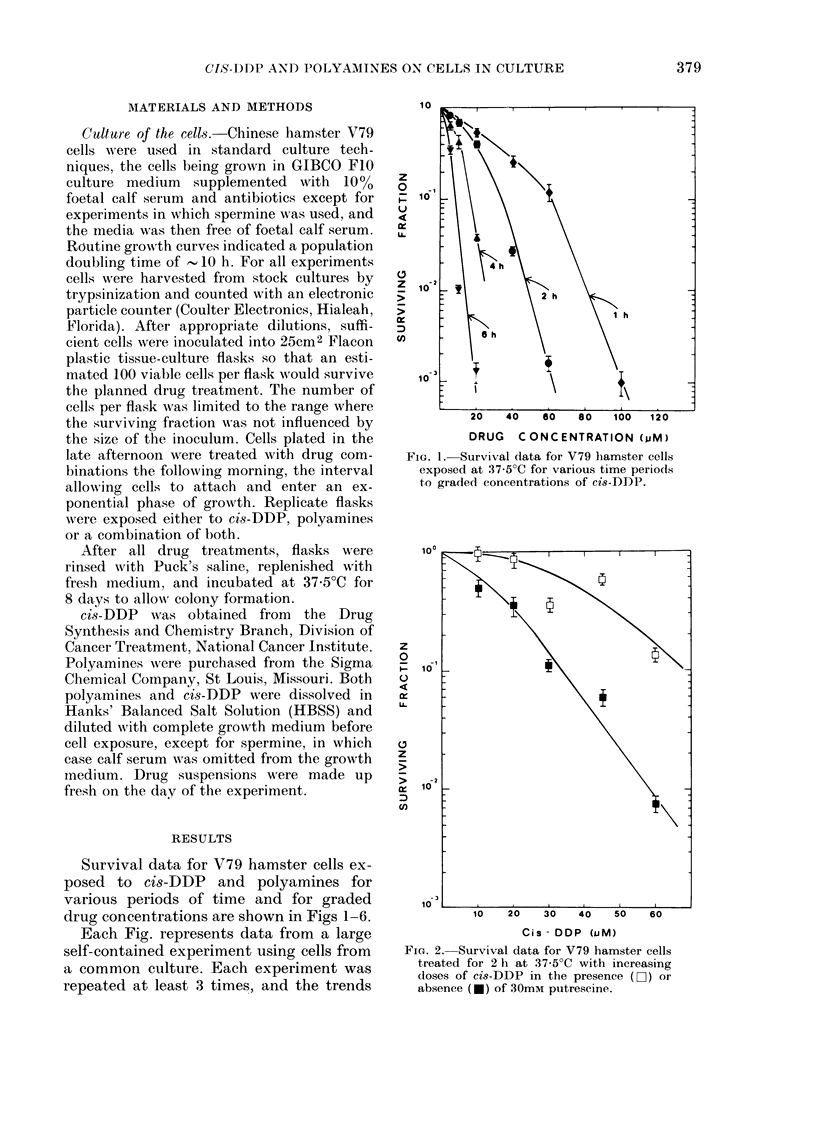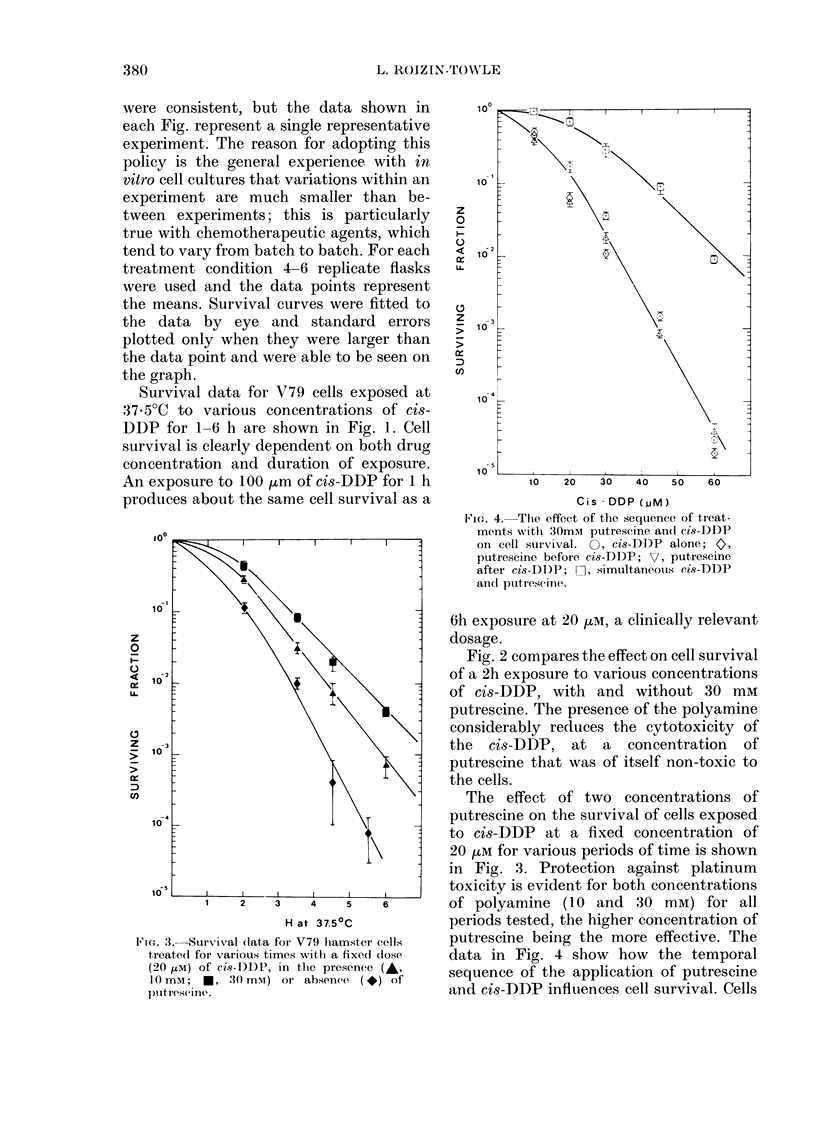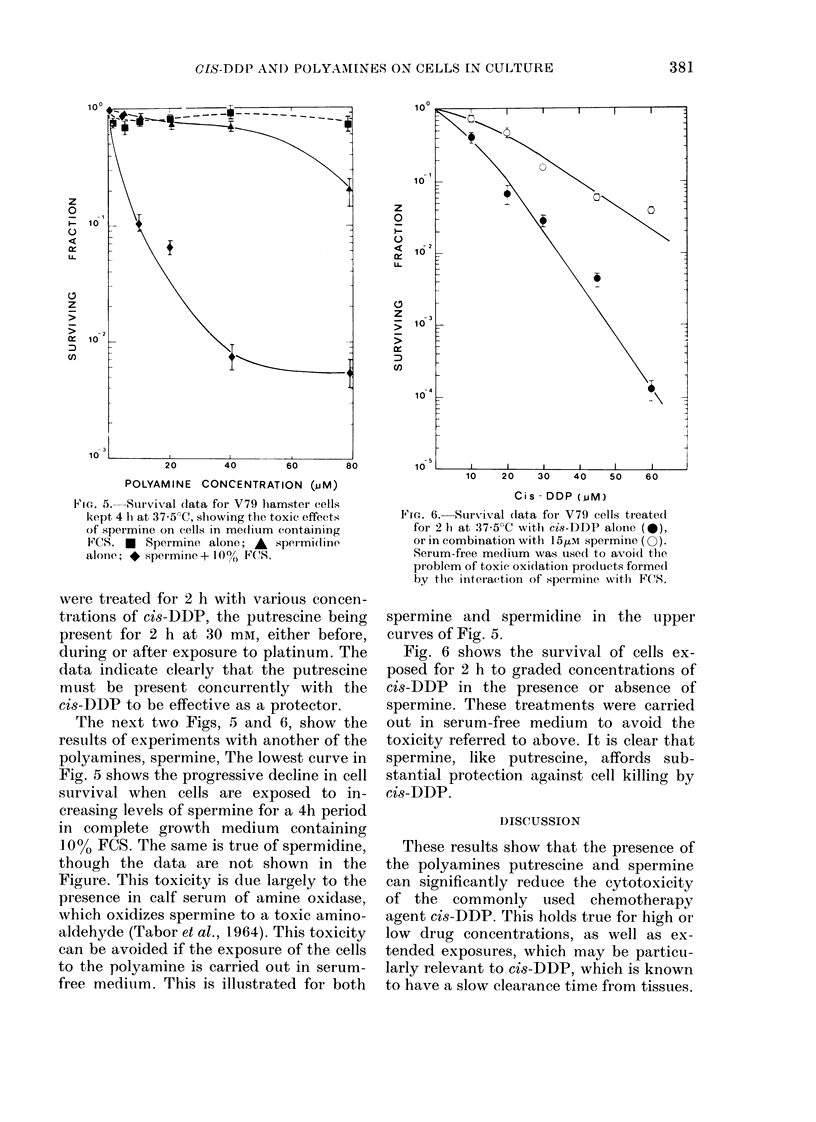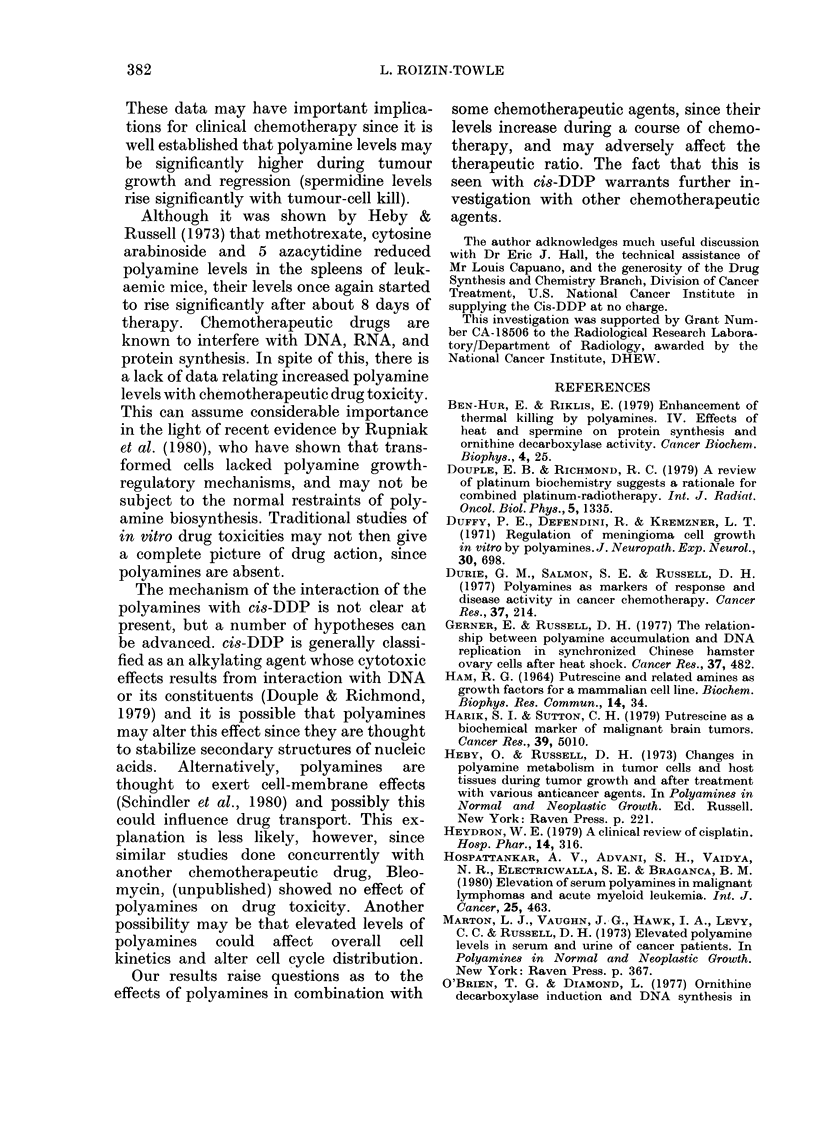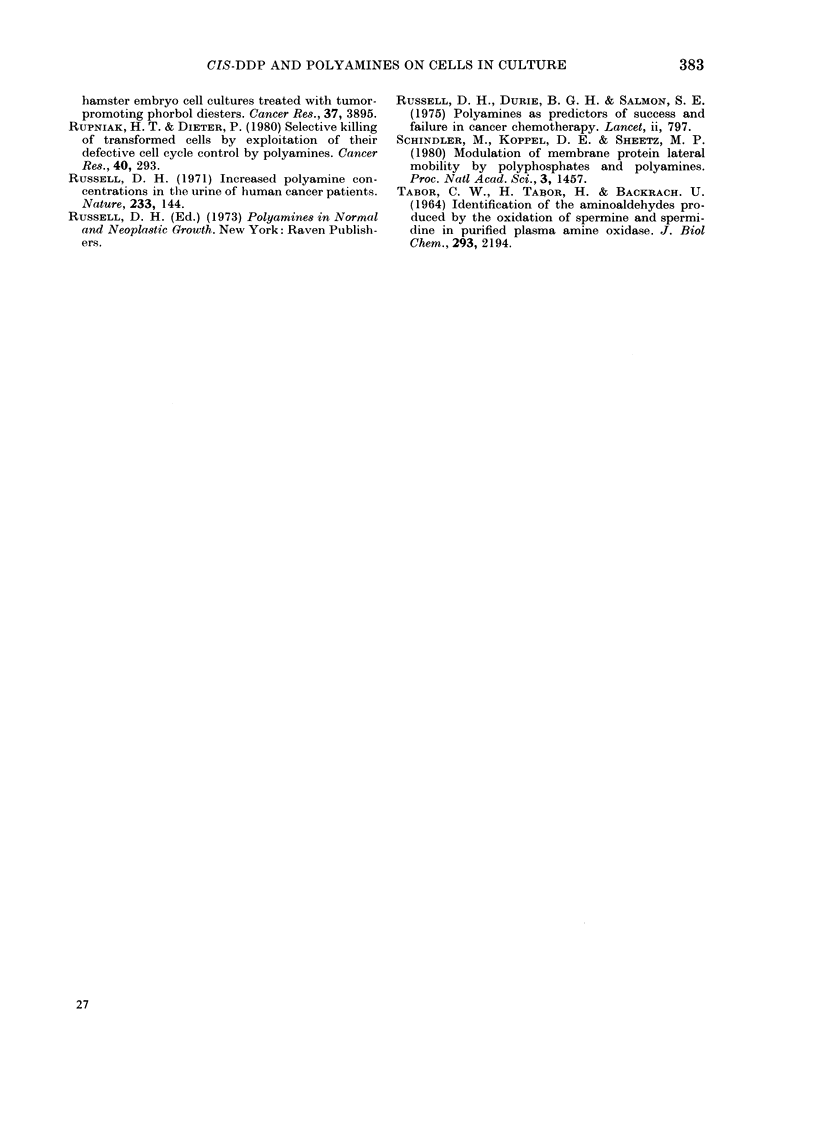# Studies with cis-diamminedichloro-platinum II and exogenous polyamines using mammalian cells in culture.

**DOI:** 10.1038/bjc.1981.58

**Published:** 1981-03

**Authors:** L. Roizin-Towle

## Abstract

Polyamines, such as putrescine and spermine, are naturally occurring substances intimately associated with normal and neoplastic growth. High levels are found in cancer patients and are associated with tumour growth. Experiments with V79 hamster cells cultured in vitro have demonstrated that the presence of putrescine and spermine can significantly reduce the cytotoxicity of the commonly used chemotherapy agent cis-DDP. These data may have clinical implications.


*Br. J. Cancer* (1981) **43**, 378

### STUDIES WITH *CIS*-DIAMMINEDICHLORO-PLATINUM II AND EXOGENOUS POLYAMINES USING MAMMALIAN CELLS IN CULTURE

L. ROIZIN-TOWLE

*From the Radiological Research Laboratory, Department of Radiology, Columbia University College of Physicians and Surgeons, New York, N.Y. 10032, U.S.A.*

Received 27 August 1980 Accepted 21 November 1980

**Summary.—Polyamines, such as putrescine and spermine, are naturally occurring substances intimately associated with normal and neoplastic growth. High levels are found in cancer patients and are associated with tumour growth. Experiments with V79 hamster cells cultured *in vitro* have demonstrated that the presence of putrescine and spermine can significantly reduce the cytotoxicity of the commonly used chemotherapy agent *cis*-DDP. These data may have clinical implications.**

POLYAMINES occur naturally in all living organisms, and are intimately associated with growth whether normal or neoplastic (Ham, 1964; Duffy *et al.*, 1971). Raised levels of polyamines are associated with cell growth, such as embryogenesis, heptatic regeneration, and lactation, as well as solid and haematological tumours (Hospattankar *et al.*, 1980). Their existence has been known for several years, but it is their function as growth promoters that has most recently raised interest as to their possible association with neoplastic processes (Russell, 1973). The findings, first by Russell (1971) and then others (Harik & Sutton, 1979; Marton *et al.*, 1973) that polyamine levels are raised in the urine and sera of cancer patients, have singled them out as potential biochemical markers for cancer detection. The fluctuations in levels of polyamines found during malignancy reflect pathological changes, and variations in their concentrations may be used to determine a patient's response to radiation or chemotherapy (Durie *et al.*, 1977; Russell *et al.*, 1975). The presence of polyamines and their regulating enzymes during the cancer process appear to be intimately related. This is further supported by the work of O'Brien & Diamond (1977) who showed that increased levels of polyamines were intrinsic to tumour promotion *in vitro*, and suggested that normal and transformed cells differ in their control of polyamine biosynthesis.

Among the naturally occurring polyamines, putrescine, spermine and spermidine are best known. Their increase during growth and differentiation usually precede increases in RNA, DNA and protein synthesis. Putrescine, in elevated amounts, appears to be related to the growth fraction of a tumour and elevated levels of spermidine to the cell-loss factor.

The fact that polyamines can influence cancer therapy has already been shown with hyperthermia, where their presence increases heat-mediated cell death (Ben Hur & Riklis, 1979; Gerner & Russell, 1977). It seemed relevant and pertinent, therefore, to ask whether the polyamines could likewise modify the action of antineoplastic chemotherapy agents. *cis*-Diamminedichloroplatinum was chosen for this study, a drug commonly used in clinical practice for the treatment of various testicular and bladder carcinomas, as well as tumours of the head and neck (Heydron, 1979).

### MATERIALS AND METHODS

*Culture of the cells.*—Chinese hamster V79 cells were used in standard culture techniques, the cells being grown in GIBCO F10 culture medium supplemented with 10% foetal calf serum and antibiotics except for experiments in which spermine was used, and the media was then free of foetal calf serum. Routine growth curves indicated a population doubling time of ~10 h. For all experiments cells were harvested from stock cultures by trypsinization and counted with an electronic particle counter (Coulter Electronics, Hialeah, Florida). After appropriate dilutions, sufficient cells were inoculated into 25cm$^2$ Flacon plastic tissue-culture flasks so that an estimated 100 viable cells per flask would survive the planned drug treatment. The number of cells per flask was limited to the range where the surviving fraction was not influenced by the size of the inoculum. Cells plated in the late afternoon were treated with drug combinations the following morning, the interval allowing cells to attach and enter an exponential phase of growth. Replicate flasks were exposed either to *cis*-DDP, polyamines or a combination of both.

After all drug treatments, flasks were rinsed with Puck's saline, replenished with fresh medium, and incubated at 37·5°C for 8 days to allow colony formation.

*cis*-DDP was obtained from the Drug Synthesis and Chemistry Branch, Division of Cancer Treatment, National Cancer Institute. Polyamines were purchased from the Sigma Chemical Company, St Louis, Missouri. Both polyamines and *cis*-DDP were dissolved in Hanks' Balanced Salt Solution (HBSS) and diluted with complete growth medium before cell exposure, except for spermine, in which case calf serum was omitted from the growth medium. Drug suspensions were made up fresh on the day of the experiment.

### RESULTS

Survival data for V79 hamster cells exposed to *cis*-DDP and polyamines for various periods of time and for graded drug concentrations are shown in Figs 1–6.

Each Fig. represents data from a large self-contained experiment using cells from a common culture. Each experiment was repeated at least 3 times, and the trends

FIG. 1.—Survival data for V79 hamster cells exposed at 37·5°C for various time periods to graded concentrations of *cis*-DDP.

FIG. 2.—Survival data for V79 hamster cells treated for 2 h at 37·5°C with increasing doses of *cis*-DDP in the presence (□) or absence (■) of 30mM putrescine.

were consistent, but the data shown in each Fig. represent a single representative experiment. The reason for adopting this policy is the general experience with *in vitro* cell cultures that variations within an experiment are much smaller than between experiments; this is particularly true with chemotherapeutic agents, which tend to vary from batch to batch. For each treatment condition 4–6 replicate flasks were used and the data points represent the means. Survival curves were fitted to the data by eye and standard errors plotted only when they were larger than the data point and were able to be seen on the graph.

Survival data for V79 cells exposed at 37·5°C to various concentrations of *cis*-DDP for 1–6 h are shown in Fig. 1. Cell survival is clearly dependent on both drug concentration and duration of exposure. An exposure to 100 $\mu$m of *cis*-DDP for 1 h produces about the same cell survival as a 6h exposure at 20 $\mu$M, a clinically relevant dosage.

Fig. 3.—Survival data for V79 hamster cells treated for various times with a fixed dose (20 $\mu$M) of *cis*-DDP, in the presence (▲, 10 mM; ■, 30 mM) or absence (◆) of putrescine.

Fig. 4.—The effect of the sequence of treatments with 30mM putrescine and *cis*-DDP on cell survival. ○, *cis*-DDP alone; ◇, putrescine before *cis*-DDP; ▽, putrescine after *cis*-DDP; □, simultaneous *cis*-DDP and putrescine.

Fig. 2 compares the effect on cell survival of a 2h exposure to various concentrations of *cis*-DDP, with and without 30 mM putrescine. The presence of the polyamine considerably reduces the cytotoxicity of the *cis*-DDP, at a concentration of putrescine that was of itself non-toxic to the cells.

The effect of two concentrations of putrescine on the survival of cells exposed to *cis*-DDP at a fixed concentration of 20 $\mu$M for various periods of time is shown in Fig. 3. Protection against platinum toxicity is evident for both concentrations of polyamine (10 and 30 mM) for all periods tested, the higher concentration of putrescine being the more effective. The data in Fig. 4 show how the temporal sequence of the application of putrescine and *cis*-DDP influences cell survival. Cells

Fig. 5.—Survival data for V79 hamster cells kept 4 h at 37·5°C, showing the toxic effects of spermine on cells in medium containing FCS. ■ Spermine alone; ▲ spermidine alone; ◆ spermine + 10% FCS.

Fig. 6.—Survival data for V79 cells treated for 2 h at 37·5°C with *cis*-DDP alone (●), or in combination with 15μM spermine (○). Serum-free medium was used to avoid the problem of toxic oxidation products formed by the interaction of spermine with FCS.

were treated for 2 h with various concentrations of *cis*-DDP, the putrescine being present for 2 h at 30 mM, either before, during or after exposure to platinum. The data indicate clearly that the putrescine must be present concurrently with the *cis*-DDP to be effective as a protector.

The next two Figs, 5 and 6, show the results of experiments with another of the polyamines, spermine, The lowest curve in Fig. 5 shows the progressive decline in cell survival when cells are exposed to increasing levels of spermine for a 4h period in complete growth medium containing 10% FCS. The same is true of spermidine, though the data are not shown in the Figure. This toxicity is due largely to the presence in calf serum of amine oxidase, which oxidizes spermine to a toxic aminoaldehyde (Tabor *et al.*, 1964). This toxicity can be avoided if the exposure of the cells to the polyamine is carried out in serum-free medium. This is illustrated for both spermine and spermidine in the upper curves of Fig. 5.

Fig. 6 shows the survival of cells exposed for 2 h to graded concentrations of *cis*-DDP in the presence or absence of spermine. These treatments were carried out in serum-free medium to avoid the toxicity referred to above. It is clear that spermine, like putrescine, affords substantial protection against cell killing by *cis*-DDP.

### DISCUSSION

These results show that the presence of the polyamines putrescine and spermine can significantly reduce the cytotoxicity of the commonly used chemotherapy agent *cis*-DDP. This holds true for high or low drug concentrations, as well as extended exposures, which may be particularly relevant to *cis*-DDP, which is known to have a slow clearance time from tissues.

These data may have important implications for clinical chemotherapy since it is well established that polyamine levels may be significantly higher during tumour growth and regression (spermidine levels rise significantly with tumour-cell kill).

Although it was shown by Heby & Russell (1973) that methotrexate, cytosine arabinoside and 5 azacytidine reduced polyamine levels in the spleens of leukaemic mice, their levels once again started to rise significantly after about 8 days of therapy. Chemotherapeutic drugs are known to interfere with DNA, RNA, and protein synthesis. In spite of this, there is a lack of data relating increased polyamine levels with chemotherapeutic drug toxicity. This can assume considerable importance in the light of recent evidence by Rupniak *et al.* (1980), who have shown that transformed cells lacked polyamine growth-regulatory mechanisms, and may not be subject to the normal restraints of polyamine biosynthesis. Traditional studies of *in vitro* drug toxicities may not then give a complete picture of drug action, since polyamines are absent.

The mechanism of the interaction of the polyamines with *cis*-DDP is not clear at present, but a number of hypotheses can be advanced. *cis*-DDP is generally classified as an alkylating agent whose cytotoxic effects results from interaction with DNA or its constituents (Douple & Richmond, 1979) and it is possible that polyamines may alter this effect since they are thought to stabilize secondary structures of nucleic acids. Alternatively, polyamines are thought to exert cell-membrane effects (Schindler *et al.*, 1980) and possibly this could influence drug transport. This explanation is less likely, however, since similar studies done concurrently with another chemotherapeutic drug, Bleomycin, (unpublished) showed no effect of polyamines on drug toxicity. Another possibility may be that elevated levels of polyamines could affect overall cell kinetics and alter cell cycle distribution.

Our results raise questions as to the effects of polyamines in combination with some chemotherapeutic agents, since their levels increase during a course of chemotherapy, and may adversely affect the therapeutic ratio. The fact that this is seen with *cis*-DDP warrants further investigation with other chemotherapeutic agents.

The author adknowledges much useful discussion with Dr Eric J. Hall, the technical assistance of Mr Louis Capuano, and the generosity of the Drug Synthesis and Chemistry Branch, Division of Cancer Treatment, U.S. National Cancer Institute in supplying the Cis-DDP at no charge.

This investigation was supported by Grant Number CA-18506 to the Radiological Research Laboratory/Department of Radiology, awarded by the National Cancer Institute, DHEW.

### REFERENCES

BEN-HUR, E. & RIKLIS, E. (1979) Enhancement of thermal killing by polyamines. IV. Effects of heat and spermine on protein synthesis and ornithine decarboxylase activity. *Cancer Biochem. Biophys.*, **4**, 25.

DOUPLE, E. B. & RICHMOND, R. C. (1979) A review of platinum biochemistry suggests a rationale for combined platinum-radiotherapy. *Int. J. Radiat. Oncol. Biol. Phys.*, **5**, 1335.

DUFFY, P. E., DEFENDINI, R. & KREMZNER, L. T. (1971) Regulation of meningioma cell growth *in vitro* by polyamines. *J. Neuropath. Exp. Neurol.*, **30**, 698.

DURIE, G. M., SALMON, S. E. & RUSSELL, D. H. (1977) Polyamines as markers of response and disease activity in cancer chemotherapy. *Cancer Res.*, **37**, 214.

GERNER, E. & RUSSELL, D. H. (1977) The relationship between polyamine accumulation and DNA replication in synchronized Chinese hamster ovary cells after heat shock. *Cancer Res.*, **37**, 482.

HAM, R. G. (1964) Putrescine and related amines as growth factors for a mammalian cell line. *Biochem. Biophys. Res. Commun.*, **14**, 34.

HARIK, S. I. & SUTTON, C. H. (1979) Putrescine as a biochemical marker of malignant brain tumors. *Cancer Res.*, **39**, 5010.

HEBY, O. & RUSSELL, D. H. (1973) Changes in polyamine metabolism in tumor cells and host tissues during tumor growth and after treatment with various anticancer agents. In *Polyamines in Normal and Neoplastic Growth.* Ed. Russell. New York: Raven Press. p. 221.

HEYDRON, W. E. (1979) A clinical review of cisplatin. *Hosp. Phar.*, **14**, 316.

HOSPATTANKAR, A. V., ADVANI, S. H., VAIDYA, N. R., ELECTRICWALLA, S. E. & BRAGANCA, B. M. (1980) Elevation of serum polyamines in malignant lymphomas and acute myeloid leukemia. *Int. J. Cancer*, **25**, 463.

MARTON, L. J., VAUGHN, J. G., HAWK, I. A., LEVY, C. C. & RUSSELL, D. H. (1973) Elevated polyamine levels in serum and urine of cancer patients. In *Polyamines in Normal and Neoplastic Growth.* New York: Raven Press. p. 367.

O'BRIEN, T. G. & DIAMOND, L. (1977) Ornithine decarboxylase induction and DNA synthesis in hamster embryo cell cultures treated with tumor-promoting phorbol diesters. *Cancer Res.*, **37**, 3895.

RUPNIAK, H. T. & DIETER, P. (1980) Selective killing of transformed cells by exploitation of their defective cell cycle control by polyamines. *Cancer Res.*, **40**, 293.

RUSSELL, D. H. (1971) Increased polyamine concentrations in the urine of human cancer patients. *Nature*, **233**, 144.

RUSSELL, D. H. (Ed.) (1973) *Polyamines in Normal and Neoplastic Growth*. New York: Raven Publishers.

RUSSELL, D. H., DURIE, B. G. H. & SALMON, S. E. (1975) Polyamines as predictors of success and failure in cancer chemotherapy. *Lancet*, ii, 797.

SCHINDLER, M., KOPPEL, D. E. & SHEETZ, M. P. (1980) Modulation of membrane protein lateral mobility by polyphosphates and polyamines. *Proc. Natl Acad. Sci.*, **3**, 1457.

TABOR, C. W., H. TABOR, H. & BACKRACH. U. (1964) Identification of the aminoaldehydes produced by the oxidation of spermine and spermidine in purified plasma amine oxidase. *J. Biol Chem.*, **293**, 2194.